# Impact of Hispanic Ethnicity, Geography, and Insurance Status on Cardiovascular Outcomes in Patients Undergoing Percutaneous Coronary Intervention

**DOI:** 10.1016/j.jacadv.2025.101723

**Published:** 2025-04-26

**Authors:** Revathy Sampath-Kumar, Ehtisham Mahmud, Vachaspathi Palakodeti, Lawrence Ang, Belal Al Khiami, Anna Melendez, Ryan Reeves, Ori Ben-Yehuda

**Affiliations:** Division of Cardiovascular Medicine, University of California-San Diego, San Diego, California, USA

**Keywords:** Hispanic ethnicity, percutaneous coronary intervention outcomes, racial and ethnic disparities

## Abstract

**Background:**

Hispanics are the largest and fastest growing ethnic minority population in the United States yet are poorly represented in cardiovascular outcomes studies. UC San Diego Health is a primary percutaneous coronary intervention (PCI) center for a diverse group of patients given its proximity to Mexico and underserved rural southeast Imperial County.

**Objectives:**

The purpose of this study was to study the association between Hispanic ethnicity, geography, insurance status, and PCI outcomes.

**Methods:**

The UC San Diego Health internal National Cardiovascular Data Registry CathPCI Registry was used to obtain data on patients who underwent PCI from January 2007 to September 2022. Complications and all-cause mortality within 1-year post-PCI were assessed.

**Results:**

A total of 8,295 patients (age 66 years [IQR: 58-75 years], 72% male, 33% Hispanic ethnicity, and 30% from Imperial County) were included. Hispanics and patients from Imperial County irrespective of race or ethnicity had higher body mass index and were more likely to have diabetes, hypertension, hyperlipidemia, end-stage renal disease, and peripheral vascular disease. There was no difference in mortality rates between Hispanic and non-Hispanic Whites in the entire population. However, within Imperial County, Hispanics had significantly higher 30-day (1.4% vs 0.3% *P* = 0.02), 6-month (2.2% vs 0.8% *P* = 0.01), and 1-year (2.9% vs 0.9% *P* = 0.004) mortality rates compared to non-Hispanic Whites. Patients in Imperial County had lower 30-day (1.2% vs 1.9% *P* = 0.01), 6-month (1.9% vs 3.3% *P* < 0.001), and 1-year (2.4% vs 5% *P* < 0.001) mortality rates compared to patients outside of Imperial County. There was no difference in all-cause mortality rates by insurance status in non-Hispanic Whites. Uninsured Hispanic patients had a higher 30-day mortality rate compared to Hispanic patients who had Medicare/Medicaid or private insurance (4.5% vs 2.0% vs 1.0% *P* = 0.005). Within Imperial County, uninsured Hispanic patients had markedly higher 30-day mortality rate compared to Hispanic patients who had Medicare/Medicaid or private insurance (10.4% vs 1.6% vs 0.3% *P* < 0.001).

**Conclusions:**

In socioeconomically disadvantaged areas, Hispanic patients had worse outcomes compared to non-Hispanic Whites compounded by uninsured status. There are complex demographic disparities in PCI outcomes for Hispanic patients and those residing in border zones which need to be recognized and mitigated.

Hispanics are the largest and fastest growing ethnic minority population in the United States^1^ with a high prevalence of coronary artery disease^2^ yet are poorly represented in cardiovascular outcomes studies. They are a heterogeneous group based on country of origin, race, socioeconomic, and insurances status. Previous studies on post percutaneous coronary intervention (PCI) outcomes for Hispanic patients included a small percentage of Hispanics or grouped Hispanics with other ethnic minorities, only had in-hospital or short-term follow-up, and reported conflicting results with regard to outcomes.

Some studies found that Hispanic patients had lower risk of cardiovascular death^3^^,^^4^ and mortality post-PCI^5^^,^^6^ compared to non-Hispanic patients despite a higher burden of comorbidities. This has been described as the Hispanic mortality paradox.^7^, ^8^, ^9^, ^10^ Familism, religiosity, higher fruit and vegetable intake, and the healthy immigrant hypothesis have been implicated as potential explanations for this observation.^11^, ^12^, ^13^ Though the Hispanic mortality paradox may be biased by out-migration of Hispanics to their country of origin^14^^,^^15^ and outcomes vary when disaggregated by Hispanic subgroups^16^ and birthplace.^17^

Other studies reported no difference in post-PCI or in-hospital mortality for Hispanic patients^18^, ^19^, ^20^, ^21^, ^22^, ^23^, ^24^, ^25^ with possible sex-based differences. A few studies report higher post-PCI mortality^26^, ^27^, ^28^ and worse angiographic outcomes^18^ in Hispanic patients. One study found higher post-PCI mortality in Hispanic patients during the COVID-19 pandemic but no difference prepandemic.^24^

We assessed PCI outcomes for a large Hispanic patient population at a quaternary care center in San Diego, California, USA. Our center serves as a primary PCI center for a diverse and large group of patients given our proximity to Mexico and underserved rural southeast Imperial County 150 miles east from our hospitals. Imperial county shares its eastern border with Arizona and southern border with Mexico (Central Illustration), it is designated a medically underserved area with 21% of the population living below the poverty line. The median household income in Imperial County is half that of San Diego County.^29^ Imperial County is an ethnic enclave with an 86% Hispanic and 30% foreign born population. We studied the association between Hispanic ethnicity, geography, insurance status, and PCI outcomes.

**Central Illustration:**
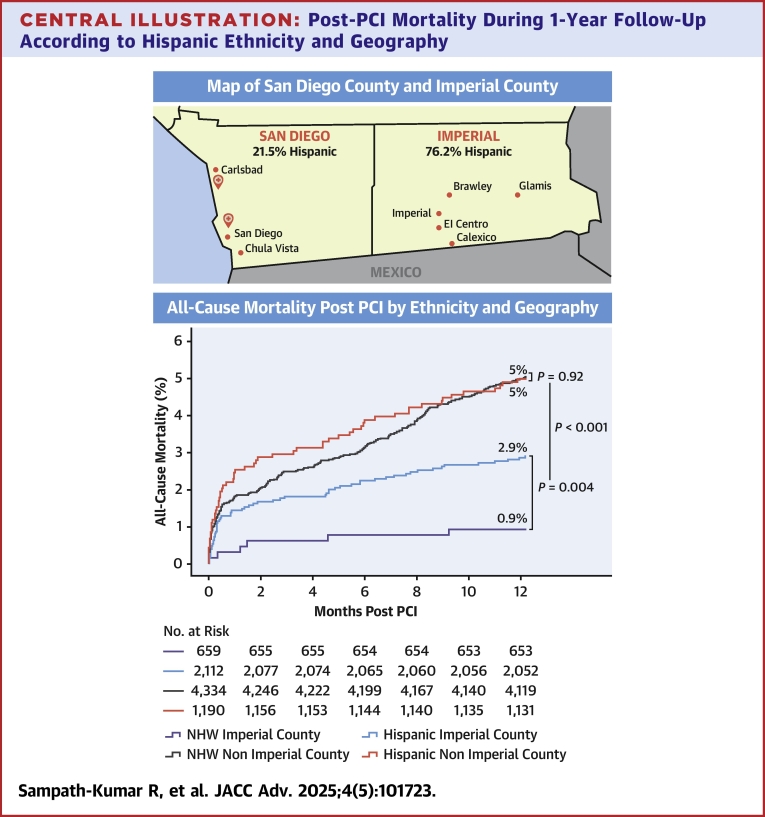
Post-PCI Mortality During 1-Year Follow-Up According to Hispanic Ethnicity and Geography (Left) Map depicting San Diego County and Imperial County in relation to Mexico and hospitals at which PCI procedures were performed. (Right) Time-to-first event curves for all-cause mortality post-PCI stratified by ethnicity and county of residence. NHW = non-Hispanic White; other abbreviation as in Figure 1.

## Methods

### Data source

The University of California-San Diego Health internal National Cardiovascular Data Registry (NCDR) CathPCI Registry was used to obtain data on patients who underwent PCI from January 2007 to September 2022 at University of California-San Diego Health. University of California-San Diego is an academic tertiary and quaternary referral hospital system that serves as a primary PCI and ST-segment elevation myocardial infarction (STEMI) receiving center for a diverse group of patients from San Diego County, Imperial County, and Mexico. PCI procedures were performed by attending interventional cardiologists with or without interventional cardiology fellows.

All-cause mortality up to 1-year post-PCI was obtained from the electronic medical record confirmed by the California Department of Public Health vital records and decedent records maintained by the Health Information Management team at UC San Diego. The Institutional Review Board of the University of California-San Diego approved the study (#809443).

### Patient selection

**Figure 1:**
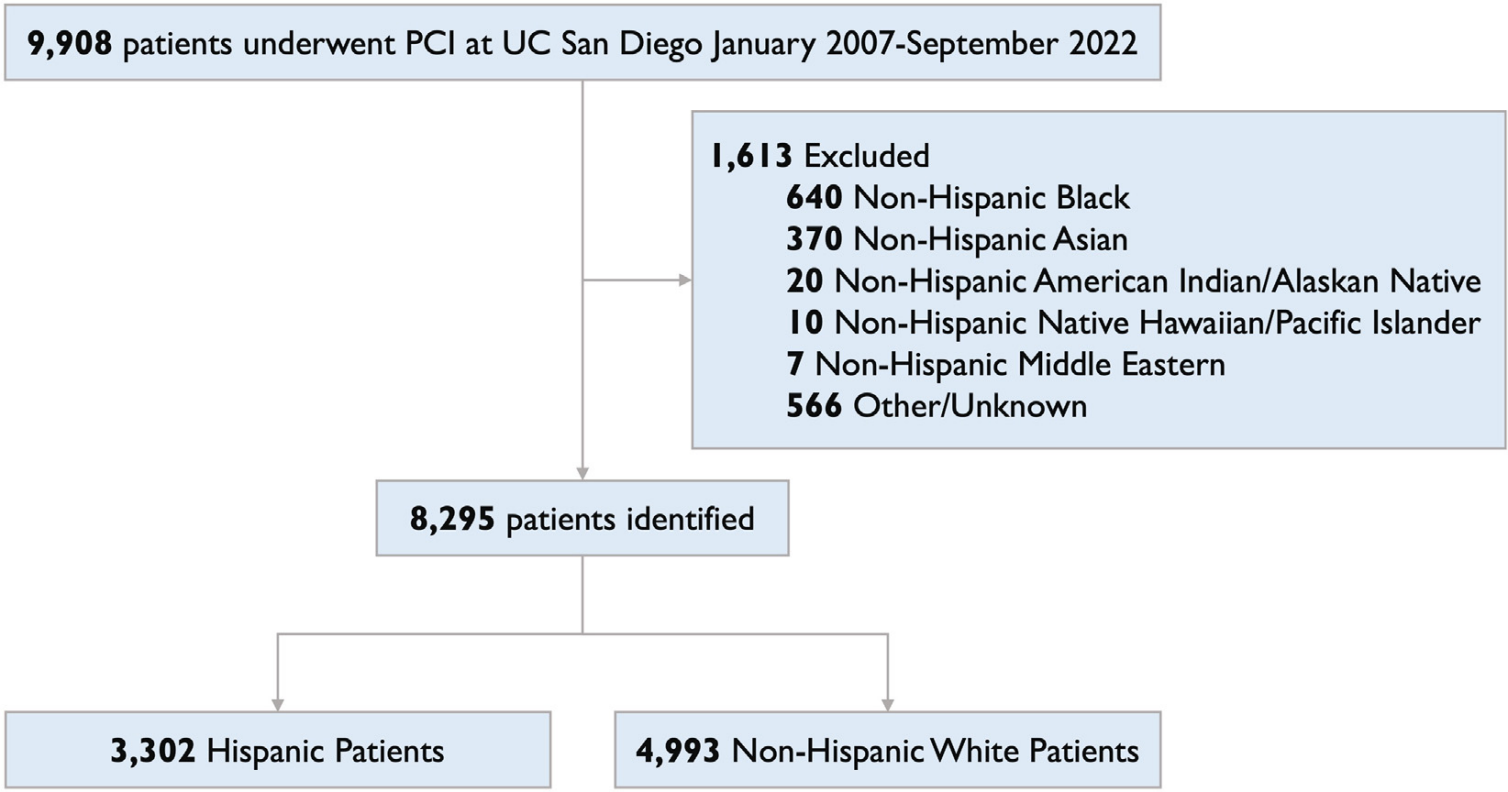
CONSORT Diagram of Patients Included in the Study Flowchart of patients included in the study. CONSORT = Consolidated Standards of Reporting Trials; PCI = percutaneous coronary intervention.

A total of 9,908 adult patients underwent PCI at University of California-San Diego Health from January 2007 to September 2022. We excluded the following self-identified non-Hispanic racial groups due to small numbers: 640 Black, 370 Asian, 20 American Indian/Alaskan Native, 10 Native Hawaiian/Pacific Islander, and 7 Middle Eastern patients. Another 566 patients had other or unknown race and ethnicity listed and were excluded. The final cohort had 8,295 patients of whom 3,302 were Hispanic and 4,993 were non-Hispanic White (Figure 1). This study followed the Strengthening the Reporting of Observational Studies in Epidemiology reporting guidelines.

### Endpoints and definitions

Outcomes assessed were complications that occurred between the procedure and hospital discharge, and all-cause mortality within 1-year post-PCI. Definitions of Hispanic Ethnicity and Hispanic Ethnicity Subgroups are from the NCDR CathPCI registry data dictionary sourced from the U.S. Office of Management and Budget Classification of Federal Data on Race and Ethnicity.^30^

Hispanic ethnicity was defined as a person that self-identified as Mexican, Puerto Rican, Cuban, South or Central American, or other Spanish culture or origin, regardless of race. NCDR version 5, which was implemented in March 2018, incorporated the Hispanic ethnicity subgroups Mexican, Puerto Rican, and Cuban, defined as having origins in any of the original peoples of these countries. Other Hispanic, Latino, or Spanish origin was designated as having origins in another Hispanic, Latino, or Spanish territory. Patients were characterized as residing in Imperial County based on their self-reported zip code associated with their home address in the electronic medical record.

### Statistical methods

Categorical data are presented as percentages and number of patients. Chi-square and Fisher exact tests were used as appropriate to compare categorical variables between groups. Continuous variables were assessed for normality using the Shapiro-Wilk test. Since none of the continuous variables met the assumption of normality, they are presented as median (IQR), and group comparisons were performed using the Mann-Whitney *U* test. Mortality was evaluated with time-to-event analysis using Kaplan-Meier curves with log-rank statistic to assess all-cause mortality at 30 days, 6 months, and 1 year. Wald chi-squared testing was used to assess the goodness-of-fit. Univariate Cox proportional hazards regression model was used to estimate HRs for all-cause mortality. Adjusted mortality analysis using baseline patient demographics, clinical presentation, and procedural characteristics determined as confounders a priori was conducted with multivariable Cox proportional hazards regression model. The proportional hazards assumption was assessed using a time-dependent Cox model and was met for 30-day and 6-month mortality. The assumption was violated for 1-year mortality but was met when the analysis was restricted to the 6-month to 1-year period. Therefore, patients who died before 6 months were censored, and Cox regression was conducted for this time frame. Propensity score matching was used to compare mortality between Hispanic and non-Hispanic White patients. Propensity scores were estimated using logistic regression with age, sex, body mass index (BMI), smoking status, hypertension (HTN), hyperlipidemia (HLD), prior myocardial infarction (MI), prior PCI, DM, acute coronary syndrome, and insurance status as covariates. Nearest neighbor matching with a caliper of 0.01 was used to create matched pairs. Analyses were performed using IBM SPSS Statistics for MacOS, version 29 (IBM Corp), a 2-sided *P* value < 0.05 was considered statistically significant.

## Results

### Patient characteristics

We included 8,295 patients, 3,302 patients of Hispanic ethnicity and 4,993 non-Hispanic White patients. Of the patients who identified as Hispanic ethnicity, 2,356 (71.4%) identified as other race, 824 (25.0%) identified as White race, 89 (2.7%) identified as American Indian/Alaskan Native race, 18 (0.5%) identified as Black race, and 15 (0.5%) identified as Asian race. Data on Hispanic subgroups were available for 1,086 Hispanic patients. Of these patients, 381 (35.1%) identified as Mexican or Mexican-American, 11 (1%) identified as Puerto Rican, 1 (0.1%) identified as Cuban, and 693 (63.8%) identified as other Hispanic, Latino, or Spanish origin. Overall, 2,771 (33.4%) patients resided in Imperial County. Within San Diego County, 21.5% of patients were of Hispanic ethnicity, and within Imperial County, 76.2% of patients were of Hispanic ethnicity.

Patient characteristics for the entire population are shown in Table 1. The median age was 66 (IQR: 58-75 years) with 27.6% female patients. Patients of Hispanic ethnicity were younger, had a greater percentage of females, and had higher BMI compared to non-Hispanic White patients. Hispanics were less likely to have prior coronary artery bypass graft surgery, prior PCI, prior MI, or family history of early coronary artery disease. Hispanic patients were more likely to have congestive heart failure, HTN, HLD, diabetes mellitus (DM), peripheral vascular disease (PVD), and end-stage renal disease (ESRD) on dialysis. Hispanic patients were less likely to smoke or have cerebrovascular disease. Hispanic patients were more likely to have Medicare or Medicaid compared to non-Hispanic White patients who were more likely to have private insurance. There was no difference in uninsured rates between Hispanic and non-Hispanic White patients (4.7% vs 4.1% *P* = 0.183).

**Table 1:** Baseline Characteristics

	Total (N = 8,295)	Hispanic (n = 3,302)	Non-Hispanic White (n = 4,993)	*P* Value
Age, y	66 (58-75)	65 (57-74)	67 (59-76)	<0.001
Sex				<0.001
Female	2,287 (27.6%)	1,015 (30.7%)	1,272 (25.5%)	
Male	6,005 (72.4%)	2,286 (69.3%)	3,719 (74.5%)	
BMI	28.19 (25.18-32.07)	29.04 (25.82-32.71)	27.68 (24.77-31.50)	<0.001
Prior CABG	1,146 (13.8%)	378 (11.4%)	768 (15.4%)	<0.001
Prior PCI	3,506 (42.3%)	1,316 (39.9%)	2,190 (43.9%)	<0.001
Prior MI	2,617 (31.5%)	998 (30.2%)	1,619 (32.4%)	0.035
Family history of premature CAD	1,304 (15.7%)	379 (11.5%)	925 (18.5%)	<0.001
CHF	2,119 (25.5%)	895 (27.1%)	1,224 (24.5%)	0.008
Dyslipidemia	6,963 (83.9%)	2,815 (85.3%)	4,148 (83.1%)	0.008
Hypertension	7,089 (85.5%)	2,955 (89.5%)	4,134 (82.8%)	<0.001
Smoker	2,712 (32.7%)	906 (27.4%)	1,806 (36.2%)	<0.001
Diabetes	3,685 (44.4%)	2,049 (62.1%)	1,636 (32.8%)	<0.001
Chronic lung disease	755 (9.1%)	182 (5.5%)	573 (11.5%)	<0.001
Peripheral vascular disease	1,035 (12.5%)	455 (13.8%)	580 (11.6%)	0.004
Cerebrovascular disease	1,077 (13.0%)	394 (11.9%)	683 (13.7%)	0.02
ESRD on dialysis	427 (5.1%)	300 (9.1%)	127 (2.5%)	<0.001
Insurance payer				<0.001
Private insurance	3,689 (44.5%)	1,069 (32.4%)	3,178 (63.6%)	
Medicare/Medicaid	359 (4.3%)	2,078 (62.9%)	1,611 (32.3%)	
None	4,247 (51.2%)	155 (4.7%)	204 (4.1%)	
Clinical presentation				<0.001
STEMI	1,095 (13.2%)	412 (12.5%)	683 (13.7%)	
NSTEMI	1,874 (22.6%)	762 (23.1%)	1,112 (22.3%)	
Unstable angina	1,036 (12.5%)	345 (10.4%)	691 (13.8%)	
Stable angina	2,971 (35.8%)	1,259 (38.1%)	1,712 (34.3%)	
Atypical chest pain	181 (2.2%)	61 (1.8%)	120 (2.4%)	
Asymptomatic	595 (7.2%)	246 (7.5%)	349 (7.0%)	
Other PCI indication	543 (6.5%)	217 (6.6%)	326 (6.5%)	
Angiographic findings				0.016
1VD not LM	6,546 (78.9%)	2,561 (77.6%)	3,985 (79.8%)	
2VD not LM	871 (10.5%)	387 (11.7%)	484 (9.7%)	
3VD not LM	77 (0.9%)	33 (1.0%)	44 (0.9%)	
LM+1VD	110 (1.3%)	40 (1.2%)	70 (1.4%)	
LM+2VD	37 (0.4%)	12 (0.4%)	25 (0.5%)	
LM+3VD	7 (0.1%)	3 (0.1%)	4 (0.1%)	
LM	70 (0.8%)	39 (1.2%)	31 (0.6%)	
Branch disease	576 (6.9%)	227 (6.9%)	349 (7.0%)	
CABG during admission	60 (0.7%)	16 (0.5%)	44 (0.9%)	0.037
Access site				0.011
Femoral	5,149 (62.1%)	2,108 (63.8%)	3,041 (60.9%)	
Radial	3,128 (37.7%)	1,188 (36.0%)	1,940 (38.9%)	
Fluoroscopy time (min)	17.9 (12.2-26.9)	17.6 (12.3-26.3)	18.2 (12.1-27.3)	0.166
Contrast volume (mL)	180 (130-240)	180 (130-240)	180 (130-240)	0.801
Door to reperfusion (min)	71 (58-94)	67 (54-86)	72 (60-96)	0.035
Days of admission	2 (1-3)	1 (1-3)	2 (1-3)	0.368
Cardiogenic shock	171 (2.1%)	53 (1.6%)	118 (2.4%)	0.017
Vasopressor	118 (1.4%)	48 (1.5%)	70 (1.4%)	0.846
MCS	447 (5.4%)	163 (4.9%)	284 (5.7%)	0.138
Cardiac arrest	142 (1.7%)	43 (1.3%)	99 (2.0%)	0.019
Any complication	685 (8.3%)	265 (8.0%)	420 (8.4%)	0.531
Any bleeding complication	371 (4.5%)	145 (4.4%)	226 (4.5%)	0.450
Any thrombotic complication	253 (3.1%)	96 (2.9%)	157 (3.1%)	0.539
Periprocedural medications				
LMWH	355 (4.3%)	168 (5.1%)	187 (3.7%)	0.003
Unfractionated heparin	5,373 (64.8%)	2,136 (64.7%)	1,237 (64.8%)	0.894
Aspirin	8,041 (96.9%)	3,212 (97.3%)	4,829 (96.7%)	0.148
Bivalirudin	5,228 (63.0%)	2,028 (61.4%)	3,200 (64.1%)	0.014
GP IIb/IIIa inhibitors	961 (11.6%)	328 (9.9%)	633 (12.7%)	<0.001
Clopidogrel	5,977 (72.1%)	2,509 (76.0%)	3,468 (69.5%)	<0.001
Prasugrel	626 (7.5%)	248 (7.5%)	378 (7.6%)	0.919
Ticagrelor	1,585 (19.1%)	596 (18.0%)	989 (19.8%)	0.046
Cangrelor	474 (5.7%)	168 (5.1%)	306 (6.1%)	0.046
Thrombolytics	230 (2.8%)	157 (4.8%)	73 (1.5%)	<0.001
Discharge medications				
ACE	3,839 (46.3%)	1,575 (47.7%)	2,264 (45.3%)	0.035
ARB	1,684 (20.3%)	758 (23.0%)	926 (18.5%)	<0.001
Aspirin	7,815 (94.2%)	3,131 (94.8%)	4,684 (93.8%)	0.054
Beta-blocker	6,308 (76.0%)	2,532 (76.7%)	3,776 (75.6%)	0.270
Statin	7,678 (92.6%)	3,104 (94.0%)	4,574 (91.6%)	<0.001
Nonstatin	1,034 (12.5%)	352 (10.7%)	682 (13.7%)	<0.001
Clopidogrel	5,946 (71.7%)	2,412 (73.0%)	3,534 (70.8%)	0.025
Prasugrel	693 (8.4%)	277 (8.4%)	416 (8.3%)	0.927
Ticagrelor	1,405 (16.9%)	531 (16.1%)	874 (17.5%)	0.091
PCSK9 inhibitor	47 (0.6%)	10 (0.3%)	37 (0.7%)	0.009

Values are n (%).

ACE = angiotensin-converting enzyme inhibitors; ARB = angiotensin receptor blockers; BMI = body mass index; CABG = coronary artery bypass graft; CAD = coronary artery disease; CHF = congestive heart failure; ESRD = end-stage renal disease; GP IIb/IIIa = glycoprotein IIb/IIIa; LM = left main; LMWH = low-molecular-weight heparin; MCS = mechanical circulatory support; MI = myocardial infarction; NSTEMI = non–ST-elevation myocardial infarction; PCI = percutaneous coronary intervention; STEMI= ST-elevation myocardial infarction; VD = vessel disease.

Patient characteristics for Imperial County are shown in Supplemental Table 1. Patients residing in Imperial County irrespective of race or ethnicity had higher BMI and were more likely to have HTN, HLD, DM, PVD, and ESRD on dialysis. Patients in Imperial County were less likely to be uninsured compared to patients outside of Imperial County (2.2% vs 5.4%; *P* < 0.001). Hispanic patients residing in Imperial County were older, had higher BMI, and were more likely to have HTN, HLD, or DM compared to Hispanic patients residing outside of Imperial County (Supplemental Table 2). Non-Hispanic White patients residing in Imperial County had higher BMI and were more likely to have HTN, HLD, PVD, or DM compared to White patients residing outside of Imperial County (Supplemental Table 3).

### Procedural characteristics

Procedural characteristics are shown in Table 1. Patients presented with STEMI (13.2%) non-ST-segment elevation MI (22.6%), unstable angina (12.5%), stable angina (35.8%), and most patients had non-left main one vessel disease. Hispanic patients were more likely to have left main involvement or femoral access compared to non-Hispanic White patients. Non-Hispanic White patients were more likely to present with cardiogenic shock, cardiac arrest, or have coronary artery bypass graft surgery during admission compared to Hispanic patients. Hispanic patients had lower door to reperfusion time for STEMI compared to non-Hispanic White patients. There was no difference in fluoroscopy time, contrast volume, length of stay, or procedural complications between groups.

Periprocedural and discharge medications are shown in Table 1. Hispanic patients were more likely to receive periprocedural low-molecular-weight heparin or thrombolytics. Non-Hispanic White patients were more likely to receive periprocedural bivalirudin, GP IIb/IIIa inhibitors, clopidogrel, ticagrelor, and cangrelor. At discharge, Hispanic patients were more likely to be prescribed angiotensin-converting enzyme inhibitors, angiotensin receptor blockers, statins, and clopidogrel. Non-Hispanic White patients were more likely to be prescribed nonstatins or PCSK9 inhibitors at discharge. There was no difference in ticagrelor prescription between groups.

### Unadjusted all-cause mortality post-PCI

In the entire population, there was no difference in 30-day, 6-month, or 1-year all-cause mortality rates between Hispanics and non-Hispanic Whites (Supplemental Table 4). Within Imperial County, Hispanics had significantly higher 30-day (1.4% vs 0.3%; *P* = 0.019), 6-month (2.2% vs 0.8%; *P* = 0.015), and 1-year mortality rates (2.9% vs 0.9%; *P* = 0.004) compared to non-Hispanic Whites (Central Illustration).

Patients in Imperial County had lower 30-day (1.2% vs 1.9% *P* = 0.009), 6-month (1.9% vs 3.3%; *P* < 0.001), and 1-year mortality rates (2.4% vs 5.0%; *P* < 0.001) compared to patients outside of Imperial County (Supplemental Table 5). Hispanic patients residing in Imperial County had lower post-PCI mortality rates at 30 days (1.4% vs 2.4%; *P* = 0.034), 6 months (2.2% vs 3.9%; *P* = 0.006), and 1 year (2.9% vs 5.0%; *P* = 0.002) compared to Hispanic patients living outside of Imperial County. There were no sex-based differences in mortality.

**Figure 2:**
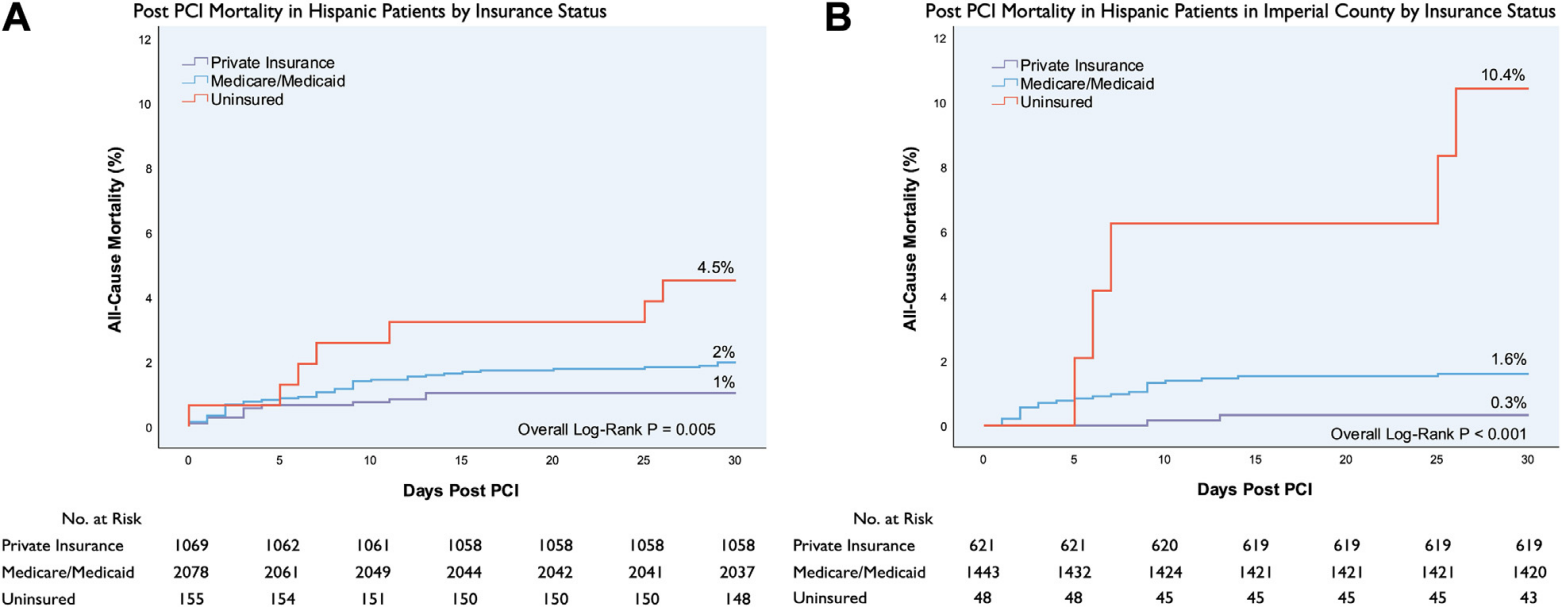
Kaplan-Meier Curves Depicting All-Cause Mortality Post-PCI by Ethnicity, County of Residence, and Insurance Status Time-to-first event curves for (A) post-PCI all-cause mortality in Hispanic patients by insurance status and (B) post-PCI all-cause mortality in Hispanic patients in Imperial County by insurance status. Abbreviation as in Figure 1.

There was no significant difference in all-cause mortality rates post-PCI by insurance status in non-Hispanic White patients (Supplemental Table 6). In the entire population, uninsured Hispanic patients had a higher 30-day all-cause mortality rate compared to Hispanic patients who had Medicare/Medicaid or private insurance (4.5% vs 2.0% vs 1.0%; *P* = 0.005) but no difference in 6-month or 1-year mortality rates (Figure 2A). Within Imperial County, uninsured Hispanic patients had even higher 30-day (10.4% vs 1.6% vs 0.3%; *P* < 0.001), 6-month (12.5% vs 2.1% vs 1.6%; *P* < 0.001), and 1-year (12.5% vs 2.8% vs 2.4%; *P* < 0.001) mortality rates compared to Hispanic patients who had Medicare/Medicaid or private insurance (Figure 2B, Supplemental Table 6).

### Adjusted all-cause mortality post-PCI

In multivariate analysis, Hispanic patients residing in Imperial County had an increased adjusted risk of all-cause mortality at 30 days (HR: 4.39; 95% CI: 1.04-18.61; *P* = 0.045) and 6 months (HR: 2.71; 95% CI: 1.07-6.91; *P* = 0.036) post-PCI compared to non-Hispanic White patients residing in Imperial County (Table 2). There was no significant difference in the adjusted risk of 6-month to 1-year mortality between groups.

**Table 2:** Risk of Adjusted and Unadjusted All-Cause Mortality Post-PCI by Hispanic Ethnicity

	Unadjusted HR (95% CI)	*P* Value	Adjusted^a^ HR (95% CI)	*P* Value
30-d all-cause mortality				
Hispanic ethnicity	1.12 (0.80-1.56)	0.526	1.19 (0.83-1.69)	0.348
Hispanic ethnicity in Imperial County	4.70 (1.12-19.68)	0.034	4.39 (1.04-18.61)	0.045
6-mo all-cause mortality				
Hispanic ethnicity	1.01 (0.77-1.31)	0.973	1.04 (0.79-1.37)	0.805
Hispanic ethnicity in Imperial County	2.96 (1.18-7.43)	0.021	2.71 (1.07-6.91)	0.036
6-mo to 1-y all-cause mortality				
Hispanic ethnicity	0.50 (0.32-0.77)	<0.001	0.49 (0.31-0.77)	0.002
Hispanic ethnicity in Imperial County	4.45 (0.59-33.81)	0.149	3.93 (0.50-30.88)	0.193

a Adjusted for age, sex, BMI, smoking status, hypertension, hyperlipidemia, diabetes, ACS, prior MI, prior PCI.

In the entire population, there was no difference in the adjusted risk of 30-day or 6-month mortality between Hispanic and non-Hispanic White patients. Hispanic patients had a lower adjusted risk of all-cause mortality from 6 months to 1 year (HR: 0.49; 95% CI: 0.31-0.77; *P* < 0.002) compared to non-Hispanic White patients.

### Propensity score-matched all-cause mortality post-PCI

The propensity score-matched cohort included 2,407 Hispanic patients and 2,407 non-Hispanic White patients. In the entire cohort, there was no difference in 30-day, 6-month, or 1-year all-cause mortality rates between Hispanics and non-Hispanic Whites (Supplemental Table 7). There was no difference in the risk of 30-day or 6-month mortality between Hispanic and non-Hispanic White patients. Hispanic patients had a lower risk of all-cause mortality from 6 months to 1 year (HR: 0.50; 95% CI: 0.29-0.87; *P* = 0.013) compared to non-Hispanic White patients.

Within Imperial County, Hispanics had significantly higher 30-day (1.7% vs 0.3%; *P* = 0.008), 6-month (2.6% vs 0.8%; *P* = 0.006), and 1-year mortality rates (3.3% vs 0.9%; *P* = 0.001) compared to non-Hispanic Whites. Hispanic patients residing in Imperial County had an increased risk of all-cause mortality at 30 days (HR: 5.58; 95% CI: 1.33-23.38; *P* = 0.019) and 6 months (HR: 3.39; 95% CI: 1.35-8.57; *P* = 0.010) post-PCI compared to non-Hispanic White patients residing in Imperial County (Supplemental Table 8). There was no significant difference in the risk of 6-month to 1-year mortality between groups.

## Discussion

In a large cohort of Hispanic patients who underwent PCI with long-term follow-up, we found disparities in outcomes for Hispanic patients compared to non-Hispanic White patients in Imperial County, which were further exacerbated by uninsured status. This study includes the largest percent of Hispanic patients and is the first to assess post-PCI outcomes in a region where the majority of the population is Hispanic. These findings are particularly relevant given the growth of the U.S. Hispanic population and the increasing number of majority Hispanic counties. The present study further emphasizes the critical role of insurance status in determining cardiovascular outcomes. Raising awareness of these disparities has the potential to inform interventions and improve cardiovascular care for Hispanic populations nationwide.

Hispanic patients in our study had higher cardiovascular risk profiles compared to non-Hispanic Whites consistent with previous studies.^31^^,^^32^ Acculturation, psychosocial factors, and lower socioeconomic status have been associated with high cardiovascular disease risk in Hispanics.^32^^,^^33^ We found that patients residing in Imperial County irrespective of race or ethnicity had higher BMI and comorbidity burden. This is likely a reflection of disadvantage from neighborhood environment limiting access to medical care, recreational options, and affordable fresh foods.^34^

Patients in Imperial County had lower post-PCI all-cause mortality rates compared to patients outside of Imperial County despite having a higher comorbidity burden and residing in a lower socioeconomic status neighborhood which has been linked to worse outcomes post-PCI.^6^^,^^35^^,^^36^ This may be driven by the lower observed uninsured rate in Imperial County. Patients who are transferred to PCI-capable hospitals are more likely to have insurance and access to medical care.^37^ Insured patients are more likely to seek community-based post-PCI care and comply with dual antiplatelet therapy. Studies have shown that government insurance and uninsured status are associated with higher mortality and readmission post-PCI.^38^, ^39^, ^40^ Our findings highlight that insurance status may be a stronger predictor of outcomes post-PCI than neighborhood of residence.

We found no difference in all-cause mortality rates post-PCI between Hispanic and non-Hispanic White patients outside of Imperial County. However, within Imperial County, Hispanic patients had significantly higher all-cause mortality rates compared to non-Hispanic White patients despite similar uninsured rates. Hispanics in Imperial County had a greater prevalence of certain cardiovascular risk factors including congestive heart failure, DM, and ESRD on dialysis compared to non-Hispanics Whites in Imperial County. However, even after multivariate adjustment, Hispanic patients in Imperial County had a higher risk of mortality at 30 days and 6 months, but not between 6 months and 1 year. Our findings challenge the previously described Hispanic mortality paradox in cardiovascular outcomes and call for a multidimensional understanding of disadvantage.

Hispanic patients residing in Imperial County had lower post-PCI mortality rates than Hispanic patients living outside of Imperial County. This is likely driven by the lower uninsured rate in Imperial County, potentially due to a larger proportion of Medicare-eligible individuals compared to San Diego County. However, the lack of data on documented immigrant status limits our interpretation. Additionally, the inclusion of only patients who underwent PCI likely introduced a selection bias toward insured patients, particularly in nonacute coronary syndrome settings.

There may also be hypothesized benefits of residing in an ethnic enclave including strength of social networks, shared language, the protective role of cultural values, and lower levels of acculturation preserving favorable diet and lifestyle practices of their country of origin.^14^^,^^32^, ^33^, ^34^^,^^41^ Hispanics patients in Imperial County had lower post-PCI mortality rates than non-Hispanic White patients outside of Imperial County. The emphasis on family within Hispanic culture may enhance social support which could contribute to better post-PCI outcomes. Higher levels of acculturation may blunt these potential benefits.^11^^,^^13^

We found no difference in all-cause mortality rates by insurance status in non-Hispanic White patients, but uninsured Hispanic patients had a higher mortality rate post-PCI compared to Hispanic patients who had Medicare/Medicaid or private insurance. Lack of insurance may disproportionately affect minoritized ethnic groups. Uninsured Hispanic patients residing in Imperial County had even higher post-PCI mortality rates at all time points highlighting the intersection of ethnic, geographic, and socioeconomic factors as it relates to outcomes.

Initiatives including STEMI cardiac care regionalization, the federal initiative to eliminate racial and ethnic health disparities, and Medicaid expansion have improved access to PCI-capable hospitals and increased PCI rates for Hispanic patients but have not translated to outcomes with lower observed benefits in minority communities.^5^^,^^42^^,^^43^ Our study highlights that complex disparities remain for Hispanic patients and those residing in border zones which need to be recognized and mitigated.

### Study Limitations

The limitations of this study include its single center, retrospective nature using registry data. We had incomplete Hispanic subgroup information as this was only collected in NCDR version 5 and were not powered to detect differences in subgroups. Socioeconomic status was inferred based on lower median household income in Imperial County and insurance status,^44^ but direct measures were not available. We did not have data on acculturation or psychosocial factors. Mortality data were tabulated from U.S. mortality statistics and did not account for foreign deaths. The COVID-19 pandemic overlapped the time frame of the study and may have impacted our results. While propensity score matching was employed to mitigate confounding, it has inherent limitations when evaluating outcomes based on ethnicity, a nonrandomizable characteristic. Ethnicity may serve as a proxy for unmeasured factors that cannot be fully controlled for in the propensity score-matched model.

## Conclusions

In this study, we found that in underserved border zones Hispanic patients had higher all-cause mortality post-PCI compared to non-Hispanic White patients. Uninsured status further exacerbated this disparity. Whereas there was no difference in post-PCI all-cause mortality by insurance status in non-Hispanic White patients.

Perspectives **COMPETENCY IN MEDICAL KNOWLEDGE:** In socioeconomically disadvantaged areas, Hispanic patients had higher mortality post-PCI compared to non-Hispanic White patients, which was further compounded by uninsured status.

**TRANSLATIONAL OUTLOOK:** Further studies with greater inclusion of Hispanic patients are needed to inform clinical practice and policies designed to mitigate these post-PCI outcome disparities.

## Funding support and author disclosures

This work was supported by an unrestricted educational grant from the Warren Family Foundation. The authors have reported that they have no relationships relevant to the contents of this paper to disclose. This research was presented in moderated poster form by Dr Revathy Sampath-Kumar at the American College of Cardiology Annual Scientific Sessions in Atlanta, Georgia, USA, on April 7, 2024.
