# Supplementary material for: Impact of Hispanic Ethnicity, Geography, and Insurance Status on Cardiovascular Outcomes in Patients Undergoing Percutaneous Coronary Intervention
Source: JACC Adv. 2025 Apr 26;4(5):101723. doi: 10.1016/j.jacadv.2025.101723 (PMC12059334; doi:10.1016/j.jacadv.2025.101723)
Supplement: Supplementary data [file mmc1.docx]

**Supplemental Table 1: Patient Characteristics Imperial County**

|  | **Total N=2,771** | **Hispanic N=2,112** | **Non-Hispanic White N=659** | **P Value** |
| --- | --- | --- | --- | --- |
| Age | 66 (59-75) | 66 (59-75) | 67 (59-74) | 0.606 |
| Sex |  |  |  | <.001 |
| Female | 837 (30.2%) | 672 (31.8%) | 165 (25.0%) |  |
| Male | 1934 (69.8%) | 1440 (68.2%) | 494 (75.0%) |  |
| BMI | 29.38 (26.18-33.10) | 29.20 (26.05-32.89) | 29.82 (26.50-33.89) | 0.018 |
| Prior CABG | 326 (11.8%) | 235 (11.1%) | 91 (13.8%) | 0.062 |
| Prior PCI | 1105 (39.9%) | 827 (39.2%) | 278 (42.2%) | 0.166 |
| Prior MI | 813 (29.3%) | 593 (28.1%) | 220 (33.4%) | 0.009 |
| Family History of Premature CAD | 385 (13.9%) | 254 (12.0%) | 131 (19.9%) | <.001 |
| CHF | 629 (22.7%) | 509 (24.1%) | 120 (18.2%) | 0.002 |
| Dyslipidemia | 2418 (87.3%) | 1846 (87.4%) | 572 (86.8%) | 0.683 |
| Hypertension | 2551 (92.1%) | 1954 (92.5%) | 597 (90.6%) | 0.11 |
| Smoker | 775 (28.0%) | 559 (26.5%) | 216 (32.8%) | 0.002 |
| Diabetes | 1674 (60.4%) | 1377 (65.2%) | 297 (45.1%) | <.001 |
| Chronic Lung Disease | 189 (6.8%) | 103 (4.9%) | 86 (13.1%) | <.001 |
| Peripheral Vascular Disease | 399 (14.4%) | 304 (14.4%) | 95 (14.4%) | 0.989 |
| Cerebrovascular Disease | 343 (12.4%) | 253 (12.0%) | 90 (13.7%) | 0.254 |
| Dialysis | 200 (7.2%) | 175 (8.3%) | 25 (3.8%) | <.001 |
| Insurance Payor |  |  |  | <.001 |
| Private Insurance | 1682 (60.7%) | 621 (29.4%) | 406 (61.6%) |  |
| Medicare/Medicaid | 62 (2.2%) | 1443 (68.3%) | 239 (36.3%) |  |
| None | 1027 (37.1%) | 48 (2.3%) | 14 (2.1%) |  |
| BMI=body mass index; CABG=coronary artery bypass graft; PCI=percutaneous coronary intervention; MI=myocardial infarction; CAD=coronary artery disease; CHF=congestive heart failure | | | | |
|  |  |  |  |  |

**Supplemental Table 2: Comparison of Hispanics in Imperial County vs Non-Imperial County**

|  | **Non-Imperial County N=1,190** | **Imperial County N=2,112** | **P Value** |
| --- | --- | --- | --- |
| Age | 62 (55-71) | 66 (59-75) | <0.001 |
| Sex |  |  | 0.085 |
| Female | 343 (28.8%) | 672 (31.8%) |  |
| Male | 846 (71.1%) | 1440 (68.2%) |  |
| BMI | 28.78 (25.46-32.43) | 29.20 (26.05-32.89) | 0.003 |
| Smoker | 347 (29.2%) | 559 (26.5%) | 0.096 |
| Hypertension | 1001 (84.1%) | 1954 (92.5%) | <.001 |
| Dyslipidemia | 969 (81.4%) | 1846 (87.4%) | <.001 |
| Family History of Premature CAD | 125 (10.5%) | 254 (12.0%) | 0.188 |
| Prior MI | 405 (34.0%) | 593 (28.1%) | <.001 |
| CHF | 386 (32.4%) | 509 (24.1%) | <.001 |
| Prior PCI | 489 (41.1%) | 827 (39.2%) | 0.275 |
| Prior CABG | 143 (12.0%) | 235 (11.1%) | 0.441 |
| Dialysis | 125 (10.5%) | 175 (8.3%) | 0.033 |
| Cerebrovascular Disease | 141 (11.8%) | 253 (12.0%) | 0.912 |
| Peripheral Vascular Disease | 151 (12.7%) | 304 (14.4%) | 0.172 |
| Chronic Lung Disease | 79 (6.6%) | 103 (4.9%) | 0.033 |
| Diabetes | 672 (56.5%) | 1377 (65.2%) | <.001 |
| Insurance Payor |  |  | <.001 |
| Private | 448 (37.6%) | 621 (29.4%) |  |
| Medicare/Medicaid | 635 (53.4%) | 1443 (68.3%) |  |
| None | 107 (9.0%) | 48 (2.3%) |  |
| BMI=body mass index; CABG=coronary artery bypass graft; PCI=percutaneous coronary intervention; MI=myocardial infarction; CAD=coronary artery disease; CHF=congestive heart failure | | | |
|  |  |  |  |

**Supplemental Table 3: Comparison of Non-Hispanic Whites in Imperial County vs Non-Imperial County**

|  | **Non-Imperial County N=4,334** | **Imperial County N=659** | **P Value** |
| --- | --- | --- | --- |
| Age | 67 (59-76) | 67 (59-74) | 0.451 |
| Sex |  |  | 0.825 |
| Female | 1107 (25.5%) | 165 (25%) |  |
| Male | 3225 (74.4%) | 494 (75%) |  |
| BMI | 27.36 (24.50-31.06) | 28.82 (26.50-33.89) | <0.001 |
| Smoker | 1590 (36.7%) | 216 (32.8%) | 0.052 |
| Hypertension | 3537 (81.6%) | 597 (90.6%) | <.001 |
| Dyslipidemia | 3576 (82.5%) | 572 (86.8%) | 0.006 |
| Family History of Premature CAD | 794 (18.3%) | 131 (19.9%) | 0.337 |
| Prior MI | 1399 (32.3%) | 220 (33.4%) | 0.573 |
| CHF | 1104 (25.5%) | 120 (18.2%) | <.001 |
| Prior PCI | 1912 (44.1%) | 278 (42.2%) | 0.352 |
| Prior CABG | 677 (15.6%) | 91 (13.8%) | 0.230 |
| Dialysis | 102 (2.4%) | 25 (3.8%) | 0.029 |
| Cerebrovascular Disease | 593 (13.7%) | 90 (13.7%) | 0.986 |
| Peripheral Vascular Disease | 485 (11.2%) | 95 (14.4%) | 0.016 |
| Chronic Lung Disease | 487 (11.2%) | 86 (13.1%) | 0.174 |
| Diabetes | 1339 (30.9%) | 297 (45.1%) | <.001 |
| Insurance Payor |  |  | 0.003 |
| Private | 2772 (64.0%) | 406 (61.6%) |  |
| Medicare/Medicaid | 1372 (31.7%) | 239 (36.3%) |  |
| None | 190 (4.4%) | 14 (2.1%) |  |
| BMI=body mass index; CABG=coronary artery bypass graft; PCI=percutaneous coronary intervention; MI=myocardial infarction; CAD=coronary artery disease; CHF=congestive heart failure | | | |
|  |  |  |  |

**Supplemental Table 4: All-Cause Mortality Post PCI by Hispanic Ethnicity and County of Residence**

|  | **Entire Population** | |  | **Imperial County** | |  |
| --- | --- | --- | --- | --- | --- | --- |
|  | **Non-Hispanic White N=4,993** | **Hispanic N=3,302** | **P Value** | **Non-Hispanic White N=659** | **Hispanic N=2,112** | **P Value** |
| 30-Day Mortality | 80 (1.6%) | 59 (1.8%) | 0.658 | 2 (0.3%) | 30 (1.4%) | 0.019 |
| 6-Month Mortality | 140 (2.8%) | 93 (2.8%) | 0.998 | 5 (0.8%) | 47 (2.2%) | 0.015 |
| 1-Year Mortality | 224 (4.5%) | 120 (3.6%) | 0.150 | 6 (0.9%) | 61 (2.9%) | 0.004 |

**Supplemental Table 5: All-Cause Mortality Post PCI by County of Residence**

|  | **Non-Imperial County N=5,524** | **Imperial County N=2,771** | **P Value** |
| --- | --- | --- | --- |
| 30-Day Mortality | 107 (1.9%) | 32 (1.2%) | 0.009 |
| 6-Month Mortality | 181 (3.3%) | 52 (1.9%) | <0.001 |
| 1-Year Mortality | 277 (5.0%) | 67 (2.4%) | <0.001 |

**Supplemental Table 6. All-Cause Mortality Post PCI by Ethnicity, County of Residence, and Insurance Status**

|  | **Medicare/Medicaid** | **Uninsured** | **Private Insurance** | **P Value** |
| --- | --- | --- | --- | --- |
| Non-Hispanic White | | |  |  |
| 30-Day Mortality | 29 (1.8%) | 4 (2.0%) | 47 (1.5%) | 0.646 |
| 6-Month Mortality | 49 (3.0%) | 5 (2.5%) | 86 (2.7%) | 0.764 |
| 1-Year Mortality | 79 (4.9%) | 6 (2.9%) | 139 (4.4%) | 0.390 |
| Hispanic | | |  |  |
| 30-Day Mortality | 41 (2.0%) | 7 (4.5%) | 11 (1.0%) | 0.005 |
| 6-Month Mortality | 56 (2.7%) | 8 (5.2%) | 29 (2.7%) | 0.195 |
| 1-Year Mortality | 74 (3.6%) | 8 (5.2%) | 38 (3.6%) | 0.582 |
| Hispanic in Imperial County | | |  |  |
| 30-Day Mortality | 23 (1.6%) | 5 (10.4%) | 2 (0.3%) | <0.001 |
| 6-Month Mortality | 31 (2.1%) | 6 (12.5%) | 10 (1.6%) | <0.001 |
| 1-Year Mortality | 40 (2.8%) | 6 (12.5%) | 15 (2.4%) | <0.001 |

**Supplemental Table 7. Propensity Score Matched All-Cause Mortality Post PCI by Hispanic Ethnicity and County of Residence**

|  | **Entire Population** | |  | **Imperial County** | |  |
| --- | --- | --- | --- | --- | --- | --- |
|  | **Non-Hispanic White N=2,407** | **Hispanic N=2,407** | **P Value** | **Non-Hispanic White N=659** | **Hispanic N=1,723** | **P Value** |
| 30-Day Mortality | 43 (1.8%) | 43 (1.8%) | 1.000 | 2 (0.3%) | 29 (1.7%) | 0.008 |
| 6-Month Mortality | 63 (2.6%) | 71 (2.9%) | 0.483 | 5 (0.8%) | 44 (2.6%) | 0.006 |
| 1-Year Mortality | 102 (4.2%) | 90 (3.7%) | 0.377 | 6 (0.9%) | 56 (3.3%) | 0.001 |

**Supplemental Table 8. Propensity Score Matched Cox Regression for All-Cause Mortality Post PCI by Hispanic Ethnicity and County of Residence**

| **PSM Cox Regression All-Cause Mortality Post PCI** | **Hazard Ratio (95% CI)** | **P Value** |
| --- | --- | --- |
| 30-Day All-Cause Mortality |  |  |
| Hispanic Ethnicity | 1.00 (0.65-1.52) | 0.995 |
| Hispanic Ethnicity in Imperial County | 5.58 (1.33-23.38) | 0.019 |
| 6-Month All-Cause Mortality |  |  |
| Hispanic Ethnicity | 1.13 (0.80-1.58) | 0.492 |
| Hispanic Ethnicity in Imperial County | 3.39 (1.35-8.57) | 0.010 |
| 6-Month to 1-Year All-Cause Mortality |  |  |
| Hispanic Ethnicity | 0.50 (0.29-0.87) | 0.013 |
| Hispanic Ethnicity in Imperial County | 4.69 (0.61-36.05) | 0.138 |
